# Enhancing the discovery of rare disease variants through hierarchical modeling

**DOI:** 10.1186/1753-6561-5-S9-S16

**Published:** 2011-11-29

**Authors:** Gary K Chen

**Affiliations:** 1 Division of Biostatistics, Department of Preventive Medicine, University of Southern California, 2001 North Soto Street, SSB 202Q, MC 9234, Los Angeles, CA 90089-9234, USA

## Abstract

Advances in next-generation sequencing technology are enabling researchers to capture a comprehensive picture of genomic variation across large numbers of individuals with unprecedented levels of efficiency. The main analytic challenge in disease mapping is how to mine the data for rare causal variants among a sea of neutral variation. To achieve this goal, investigators have proposed a number of methods that exploit biological knowledge. In this paper, I propose applying a Bayesian stochastic search variable selection algorithm in this context. My multivariate method is inspired by the combined multivariate and collapsing method. In this proposed method, however, I allow an arbitrary number of different sources of biological knowledge to inform the model as prior distributions in a two-level hierarchical model. This allows rare variants with similar prior distributions to share evidence of association. Using the 1000 Genomes Project single-nucleotide polymorphism data provided by Genetic Analysis Workshop 17, I show that through biologically informative prior distributions, some power can be gained over noninformative prior distributions.

## Background

Genome-wide association studies (GWAS) have been a powerful method for revealing common variants that confer a modest increase in disease risk in carriers. In general, the single-nucleotide polymorphisms (SNPs) that show the strongest evidence for association in GWAS do not perfectly tag the putative causal variant(s) nearby because of ancestral recombination events; therefore resequencing in these regions is necessary to resolve the precise location of the causal variant(s). Dickson et al. [1] postulated one possible explanation for why many fine-mapping efforts have failed to map a single causal SNP in the region tagged by the original genome-wide association signal: multiple rare variants (MRVs) residing on multiple haplotypes at the region of the genome-wide association signal are generating a “synthetic” association when these haplotypes share a common allele that is observed more in case subjects than in control subjects. In support of the MRV hypothesis, several investigators have recently developed a number of popular burden-type methods [2-4]. These methods are predicated on the notion that presence of or an increase in the number of mutations for a person at a particular pathway, region, gene, or any other biological unit can serve as a reasonable proxy for his/her risk of developing disease. The common theme among these methods is that the genotypes for MRVs that map to these biological units, called bins, are collapsed into a single vector of scores, a technique that can potentially improve statistical power to detect disease association. For example, in the combined multivariate and collapsing (CMC) method of Li and Leal [2], a score for an individual is assigned 1 if at least one mutation is observed across all SNPs within a bin, or 0 otherwise. The significance of a gene, for example, can then be tested by jointly modeling all bins that map within the gene using a multivariate method such as Hotelling’s multivariate *T*-test, logistic regression, or linear regression.

In this paper, I describe how I adapted the concept of the CMC method into a Bayesian variable selection algorithm with the notion that common SNPs may also contribute valuable information to nearby causal rare variants, assuming that the shared haplotype model [1] is true. The exon resequencing data set provided by the organizers of Genetic Analysis Workshop 17 (GAW17) provides an ideal opportunity for evaluating the performance of this new approach.

## Methods

Details of the simulated GAW17 data set can be found in this same issue [5]. I defined variants that had a minor allele frequency (MAF) less than 0.01 to be rare but potentially the most biologically interesting, because extremely rare mutations are expected to have the greatest deleterious effects on phenotype. Of all the SNPs provided in the data set, 73% (18,131) fall within this MAF range. For each gene, I applied the collapsing procedure, as described in the CMC method [2], by grouping rare SNPs into one of two bins defined by their predicted impact on protein (i.e., synonymous or nonsynonymous variant). Any bin with a MAF less than 0.01 after the collapsing procedure was not included for further analysis. Common SNPs, defined as those having a MAF ≥ 0.01, were not collapsed with any other SNPs in the gene. For conciseness, I use the term *variable* to define either a single common SNP or a SNP bin. The final marker panel included 7,385 variables: 1,029 bins containing collapsed rare variants and 6,356 bins containing common SNPs. I experimented with higher threshold values for bin definition (e.g., MAF = 0.05), but this strategy did not recover an appreciable number of bins from the filtering step because most genes in the data set were small and harbored private mutations. True log relative risks (denoted **β**) for each SNP are provided in the simulation answer key, which quantifies each SNP’s effect on the quantitative traits Q1 and Q2. Thus, to assess how accurately my method can recover the true values of **β** at each SNP, I constrained the analyses only to models where the outcome phenotype was either trait Q1 or Q2.

The statistical model I used was a two-level hierarchical model, described in detail by Chen and Thomas [6]. One property of a hierarchical model that is appealing when analyzing variants of low frequency, where maximum-likelihood estimates (MLEs) of association  can be highly unstable, is the ability to smooth these point estimates (and their variances) toward prior distributions defined in a second level. At the first level, I apply ordinary least-squares regression, which produces MLEs of association between a continuous trait (i.e., either Q1 or Q2) and a random set of *m* model variables. A design matrix **X** stores the variable values, and the vector **Y** stores values of Q1 or Q1 across all individuals:(1)

I define a prior distribution on **β** in Eq. (1) using the annotation information provided by GAW17. For variable *k*, **β***_k_* is distributed as a mixed-effects model, originally defined by Besag et al. [7] as:(2)

where the latent fixed effect is *π* and the random effects components are:

The **Z** matrix stores external knowledge about each of the *m* variables currently in the model. To encode my belief that deleterious mutations would have higher or lower values of **β** relative to other types of mutations, I assigned a value of 1 to the nonsynonymous mutation in the second column (after reserving the first column as the intercept) of the *m* × 2 design matrix **Z** and a value of 0 for any other SNP category. The term *π*, estimated using ordinary linear regression, relates the magnitude of **β** in Eq. (1) to values in **Z**. Furthermore, to encode my belief that mutations within the same gene should have similar effects on disease, I specified an indicator encoding whether predictor *k* and any other model variables are in the same gene by means of a *k* × *k* adjacency matrix **A**. Specifically, the parameter  stores the mean of the MLE  from the first level, taken across neighbors of variable *k* (i.e., all other variables that are in the same gene) defined by means of **A**. The variance term *τ*^2^ is inversely scaled by *v_k_*, the number of neighbors of *k* to weight the uncertainty about *τ*^2^. Finally, *θ_k_* soaks up any remaining variation in the second level of the model through the variance term *σ*^2^.

A posterior density is defined on the basis of the likelihood and normal density function corresponding to the first (Eq. (1)) and second (Eq. (2)) levels of the hierarchical model. I use the product of this density function and a model transition function as the objective function of a reversible jump Monte Carlo Markov chain (MCMC) algorithm to stochastically explore the search space, fitting all possible sets of model variables to the data. The model transition kernel itself is informed through empirical Bayes estimates of the hyperparameters (e.g., *π*), so that regions of the search space that have strong empirical support and prior evidence are prioritized. Further details on how the variable selection algorithm works can be found in Chen and Thomas [6].

In the next section I present results between a more conventional method and my proposed method. The first method is an ordinary least-squares regression between the quantitative trait (i.e., Q1 or Q2) and each vector of variable scores, which I denote as the MLE method. This approach is equivalent to a conventional genome-wide association scan, testing for marginal effects. I compared this to four variations of the multivariate MCMC method. Specifically, I varied the degree of informativeness in the prior distribution by modifying the definition of the matrices **A** and **Z**. The most informative prior distribution (denoted FULL) stores both gene membership and SNP mutation type information in the **A** and **Z** matrices, respectively. In the second variant of the prior distribution (denoted **Z** only), I removed gene membership information so that matrix **A** was simply the identity matrix. Conversely, in the third variant of the prior distribution (denoted **A** only), I removed mutation class information so that the **Z** matrix included only the intercept. The last variant of the prior distribution (denoted UNINF) includes both the uninformative **Z** and **A** matrices and is equivalent to a the ridge style prior distribution (i.e., **β** ~ *N*(0, *σ*^2^ )).

For each of the MCMC analyses, I sampled 2 million realizations from the posterior distribution, retaining statistics on only the last million realizations to minimize any correlation to the initial parameter values. Run time on a 2-GHz Xeon processor was approximately 8 h. I verified that the retained statistics reached convergence by comparing their distributions across multiple chains using a nonsignificant *p*-value extracted from the Kolmogorov-Smirnov test.

To quantify evidence for any specific variable (either common SNP or SNP bin), I empirically estimated Bayes factors (BFs) for each variable by dividing the posterior odds by the prior odds, as described by Chen and Thomas [6]. BFs quantify the increase in evidence for a hypothesis (in this case, inclusion of a variable into the model) in light of observed data relative to a prior hypothesis [8].

## Results

Table 1 lists the posterior estimates of the various hyperparameters of the hierarchical model under the FULL prior distribution specification. For either of the two quantitative traits, the residual variance (*τ*^2^) in the random effects component was smaller than the residual variance from the fixed effects component (*σ*^2^), indicating a good fit between the gene-membership prior distribution and the observed data. The posterior estimates for the prior mean (*π*) indicate a slightly positive correlation (0.03) between disease risk and presence of a nonsynonymous mutation, although the evidence is weak considering the large standard errors (0.06).

**Table 1:** Posterior estimates of hyperparameters

Parameter	Trait Q1	Trait Q1
*τ*^2^	0.006	0.006
*σ*^2^	0.01	0.01
*π* (SE)	0.03 (0.06)	0.02 (0.06)

As alluded to earlier, hierarchical modeling shrinks unstable MLEs toward means informed through either informative or noninformative prior distributions. I considered two metrics that measure the accuracy of a method’s estimation of the true effect size: the mean coverage rate (MCR) and the root mean-square error (RMSE). I defined the MCR as the proportion across all causal SNPs and simulation replicates where the true value of **β** falls within the 95% confidence interval of the estimator. Thus a perfect estimator would have a value of 1. Hierarchical modeling achieved an MCR of 0.91 under the Q1 disease model, in contrast to an MCR of 0.56 when applying maximum likelihood. The second metric I considered, RMSE, is calculated by taking the square root of the average squared difference (also taken across all markers and replicates) between the estimated and true values of **β**. A smaller value of the RMSE indicates a more precise estimation of the true effect size. Under the Q1 disease model, the RMSE for the hierarchical model was 0.17, whereas for the maximum-likelihood model it was 0.38. When Q2 was the disease model, the RMSE and MSE were similar (within ±0.01), approximately 0.17 and 0.94, respectively, regardless of which method was used. Table 2 presents a list of causal variables under the Q1 disease model, indicating that several SNPs at the *FLT1* gene were poorly estimated using maximum likelihood.

**Table 2:** Accuracy of estimates of β for trait Q1 between maximum-likelihood estimate (MLE) and hierarchical modeling (HM) estimates

Variable	Mean square error		Mean coverage rate^a^	
	
	MLE	HM	MLE	HM
C1S6521	0.196	0.046	0.68	0.94
C13S398	0.069	0.036	0.90	0.93
C13S515	0.300	0.029	0.07	0.92
C13S522	0.126	0.037	0.06	0.71
HFE, nonsynonymous	0.218	0.033	0.63	0.98
KCTD14, nonsynonymous	0.007	0.009	0.91	0.84
C4S1878	0.102	0.024	0.7	0.95

^a^ Proportion of replicates where true **β** falls within the 95% confidence interval.

**Figure 1:**
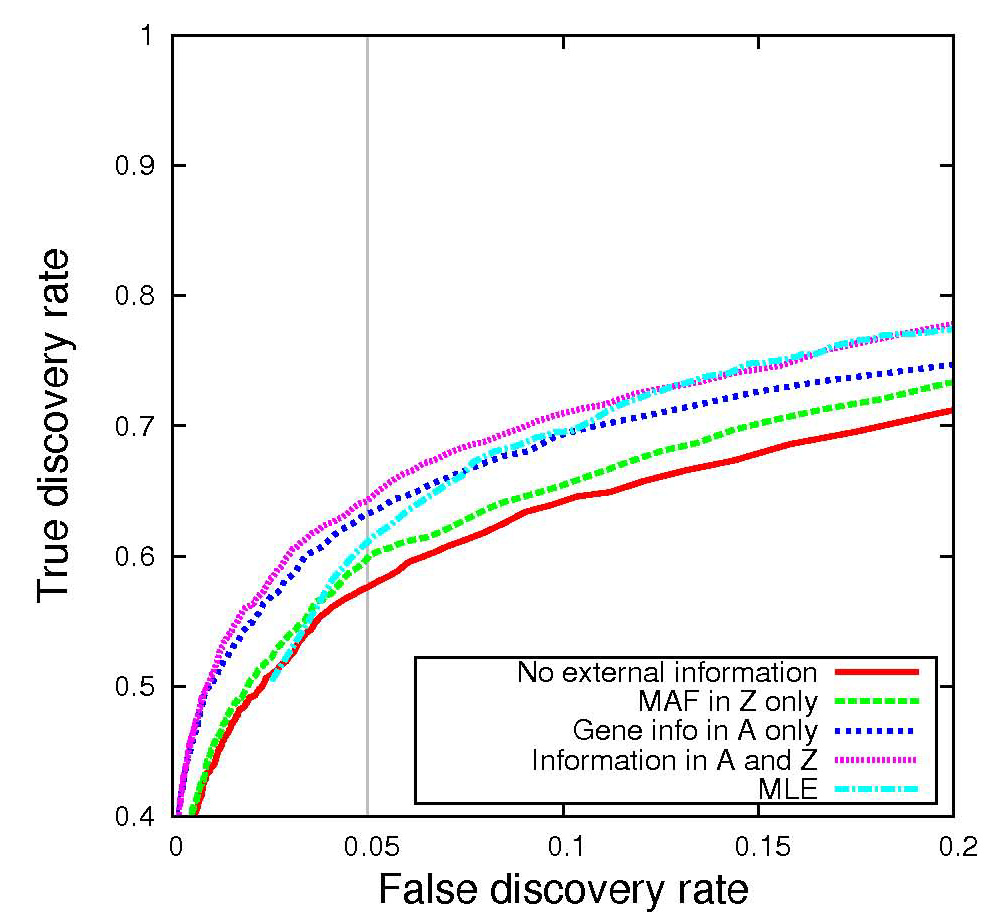
**Receiver operating characteristic curve under polygenic disease model for trait Q1.** The proportion of causal variants is plotted as a function of the proportion of noncausal variants, taken across 200 replicates.

**Figure 2:**
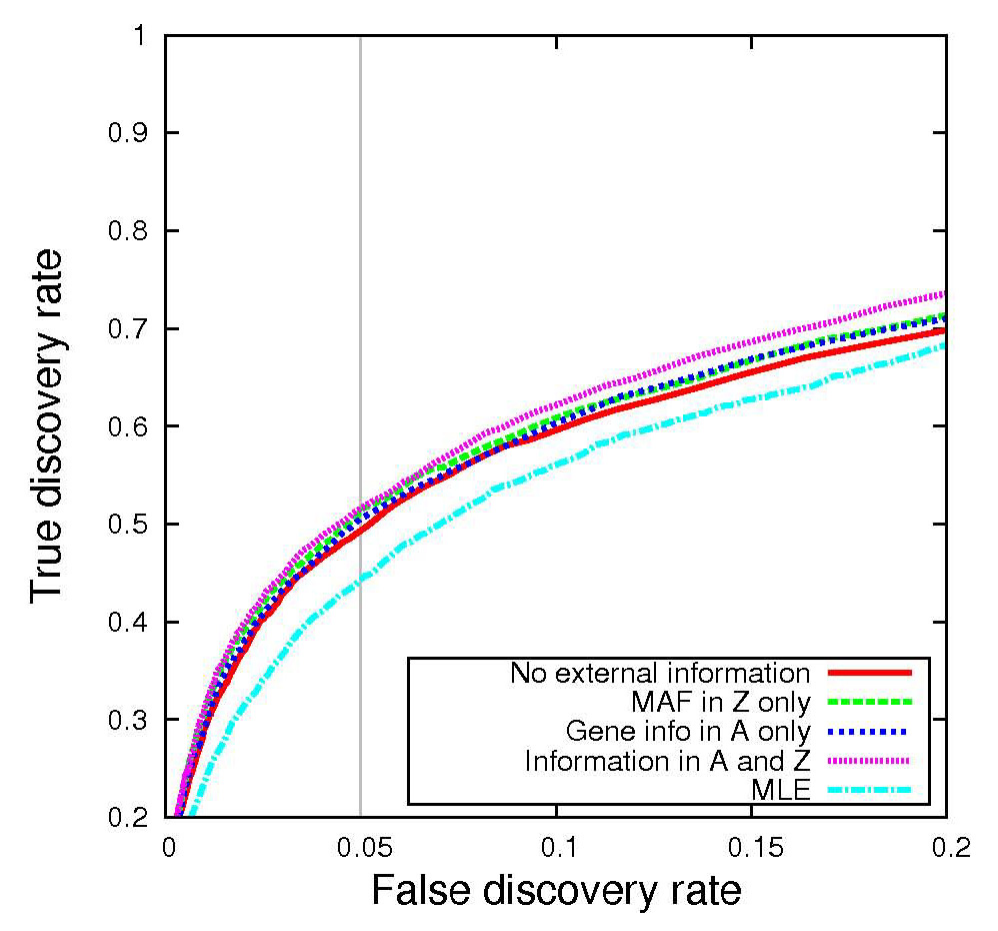
**Receiver operating characteristic curve under polygenic disease model for traitQ2. **The proportion of causal variants is plotted as a function of the proportion of noncausal variants, taken across 200 replicates.

I next evaluated the ability of the MCMC sampler to perform variable selection by comparing sensitivity and specificity across the four variants of the prior distribution. The receiver operating characteristic (ROC) curves in Figures 1 and 2 illustrate power across various false discovery rates for traits Q1 and Q2, respectively. As one might expect, introducing informative prior distributions into the model improves power to detect causal variants. Gene membership information as encoded in matrix **A** proved to be the most critical component for power overall. When Q2 was used as the outcome phenotype, the method showed greater sensitivity than the MLE method across all false discovery range (FDR) values, regardless of the prior distribution specification. Q1 performed slightly worse than the MLE at low FDRs when gene membership information was omitted from the prior specification. Table 3 summarizes the relative differences in power at FDR = 0.05 between the MLE and my approach.

**Table 3:** Relative power (in relation to the maximum-likelihood estimate) of hierarchical modeling method at FDR = 0.05

Variation	Trait Q1	Trait Q2
UNINF	0.94	1.14
**Z** only	0.98	1.17
**A** only	1.04	1.17
FULL	1.05	1.19

FDR, false discovery range.

I noted a wide range of evidence across the variables considered. Tables 4 and 5 present a comparison of BFs across the various prior specifications in the variable selection algorithm for each causal variable that was included in the analysis. Although guidelines for BF interpretation [8] deem several variables to be “barely worth mentioning” (BF range, 1 to 3), others could be considered “decisive” (BF > 100). Under Q1, evidence of association was strongest for the C13S431, C13S522, and C13S523 SNPs in the *FLT1* gene, which had more “common” MAFs of 0.02, 0.03, and 0.07, respectively. These same SNPs also had fairly large simulated odds ratios (2.1, 1.9, and 1.9, respectively), which most likely explain the improved overall performance of all the methods under the Q1 model, as shown in Figures 1 and 2, in contrast to the Q2 model, whose disease model was more challenging. The only SNP under the Q1 model that was more common than these three SNPs was C4S1878 (MAF = 0.16). A relatively moderate BF of 107 at this SNP reflects its modest simulated odds ratio of 1.1. The **A** matrix information, which helps distribute evidence of association across a gene, was advantageous for SNPs within *FLT1*. In contrast, for SNPs in other genes, the **Z** matrix, which enables variables of the same mutation type to share a common mean, improved the method’s power to detect causal variants, as seen in the higher BFs in column 2 versus column 3 in Tables 4 and 5. This observation was not too surprising, considering the fact that the simulation model considered only nonsynonymous mutations to be causal.

**Table 4:** Bayes factors for each causal variable under the Q1 trait model

Causal variable^a^	UNINF	**Z** only	**A** only	FULL
*ARNT*, nonsynonymous	1.14	1.95	1.85	3.99
C4S1884	36.33	43.31	36.12	47.65
*HIF1A*, nonsynonymous	0.58	1.14	0.57	1.22
C13S522	527.13	600.4	764.92	773.35
C1S6533	109.38	149.45	119.39	162.42
C4S1878	57.23	92.15	69.16	107.15
C14S1734	1.47	2.42	1.47	2.58
C13S431	299.20	327.49	572.87	551.06
*FLT1*, nonsynonymous	17.25	27.80	81	103.17
C13S523	998.33	999.07	999.87	999.7

^a^ Defined as either a bin of SNPs (shown with convention gene name and mutation class) or a single SNP. Only variables with MAF ≥ 0.01 were included for analyses.

**Table 5:** Bayes factors for each causal variable under the Q2 trait model

Causal variable^a^	UNINF	**Z** only	**A** only	FULL
C6S5441	53.96	77.22	65.32	88.21
*SIRT1*, nonsynonymous	22.36	34.60	23.92	34.82
C2S354	11.17	15.29	11.04	16.46
C8S442	62.14	88.72	64.60	87.26
C6S5449	64.43	85.17	73.89	94.77
*SREBF1*, nonsynonymous	60.71	83.47	60.57	85.76
*PDGFD*, nonsynonymous	49.64	71.03	49.12	70.54
C6S5426	0.87	1.48	1.25	2.10
C6S5380	212.1	264.3	210.2	263.0
*PLAT*, nonsynonymous	9.28	14.54	8.89	14.44
*VLDLR*, nonsynonymous	17.51	26.85	17.15	26.93
*BCHE*, nonsynonymous	38.11	55.60	37.50	55.62
*LPL*, nonsynonymous	1.34	2.43	1.62	2.63

^a^ Defined as either a bin of SNPs (shown with convention gene name and mutation class) or a single SNP. Only variables with MAF ≥ 0.01 were included for analyses.

## Discussion

In response to the missing heritability mystery plaguing the field of complex trait genetics, there is understandably massive interest in developing methods that can effectively investigate the relationship between rare variants and disease. In the methods described by Madsen and Browning [3] and a more recent refinement described by Price et al. [4], common SNPs are down-weighted on the assumption that their effect sizes are expected to be smaller than their rarer neighbors. Details on these approaches are found in Dering et al. [9]. A one degree of freedom test is carried out at the gene level or other biological unit rather than at the SNP level. These methods are appealing because power can be increased as a result of fewer multiple hypotheses to adjust for. I took a somewhat different approach that was closer in spirit to the CMC method [2]. Like the CMC approach, my method operates within a multivariate framework so that multiple bins within a gene can be considered; this allows one to test multiple hypotheses and to refine the signal, albeit at a statistical cost resulting from multiple comparisons. In contrast to the Madsen and Browning [3] and Price et al. [4] methods, I do not down-weight SNPs of higher frequency. In fact, I believe that if there is a shared haplotype effect among case subjects, then these common SNPs can aid in discovery of rarer neighbors through an appropriate prior specification (e.g., the **A** matrix in the hierarchical model). With any type of collapsing strategy, including mine, the choice of how bins are defined is arbitrary and some type of permutation procedure is necessary to alleviate an increase in type I error from overfitting the data. My Bayesian method, while also computationally expensive, does not involve permutation. Through Bayes model averaging and reporting of BFs, the problem of model overfitting is handled naturally. I previously demonstrated through simulations that the model is robust in light of multiple comparisons within the context of discovering interactions [6].

The results from the analyses show that in certain cases, such as when Q1 is modeled as the outcome, rare variants can make accurate estimates of effect size difficult when operating under a conventional MLE framework. Hierarchical modeling can be particularly helpful here, even if the prior distributions are not particularly informative. However, I must provide an important caveat that the method, which still operates under a standard multivariate regression framework at the first level of the model, does not appear to work particularly well when rare variants (i.e., omitting a collapsing strategy), such as singleton mutations, are directly tested; convergence problems usually emerge when the design matrix becomes numerically singular. Thus I was unable to directly evaluate the method’s performance on any one specific SNP among the extremely rare causal variants. The LASSO (least absolute shrinkage and selection operator) method [10], another flavor of penalized regression that provides variable selection, has recently been extended to allow one to directly test any rare variant by defining bins (e.g., genes) that relax the global penalization parameter [11]. Although my approach is more limited in this sense, my model allows the investigator to include an arbitrary number of prior knowledge sources through columns in a **Z** matrix, as demonstrated in the sensitivity analyses presented in Figures 1 and 2. I found that defining a richer prior distribution on **β** based on biology could indeed improve power to detect variants. On closer inspection, I learned that mutation type (synonymous vs. nonsynonymous) information was more beneficial than gene-membership information for most of the SNP bins, but the opposite was true for the *FLT1* gene. Thus I recommend providing as much external knowledge as possible in the model (e.g., adding additional columns in **Z**). Because my method is based on empirical Bayes estimates, it is robust to poor specification of the prior distribution, because this only leads to increased uncertainty (modeled in the prior variances *τ*^2^ and *σ*^2^), asymptotically reducing the prior distribution on **β** to a ridge prior distribution.

Clearly, there is a need to develop methods to effectively mine the data for rare variants that confer disease risk. I am optimistic that my approach is more effective than other methods in many cases, but it does have the same limitations as those shared by collapsing-style methods, particularly the strong assumption that effect sizes will point in the same direction among SNPs inside a bin. I am considering other variations of the hierarchical model that might more flexibly accommodate this type of heterogeneity. One appealing idea is to include a new stochastic layer into the algorithm that randomly groups SNPs into bins (and consequently compresses the **A** and **Z** matrices accordingly). My method currently permits one to perform a global test of association (i.e., are any rare variants associated?) by testing fixed bins. An important property of enabling flexibility in bin assignment is that one can additionally perform local tests of association (i.e., how often does this SNP appear in any bin?).

## Conclusions

I have presented a computationally efficient Bayesian method that simultaneously provides additional power to discover rare disease variants and enhances estimation of true effect sizes. Users interested in the algorithm can download C++ source code and binaries from my website (http://www-hsc.usc.edu/~garykche/).

## Competing interests

I have no competing interests to declare.

## Authors’ contributions

GKC conceived of the study, carried out the statistical analyses, and drafted the manuscript.

## References

[B1] Dickson SP Wang K Krantz I Hakonarson H Goldstein DB Rare variants create synthetic genome-wide associations PLoS Biol 1000 8 e294 10.1371/journal.pbio.1000294 PMC2811148 20126254

[B2] Li B Leal SM Methods for detecting associations with rare variants for common diseases: application to analysis of sequence data Am J Hum Genet 2008 83 311 321 10.1016/j.ajhg.2008.06.024 18691683 PMC2842185

[B3] Madsen BE Browning SR A groupwise association test for rare mutations using a weighted sum statistic PLoS Genet 2009 5 e1000384 10.1371/journal.pgen.1000384 19214210 PMC2633048

[B4] Price AL Kryukov GV de Bakker PI Purcell SM Staples J Wei LJ Sunyaev SR Pooled association tests for rare variants in exon-resequencing studies Am J Hum Genet 2010 86 832 838 10.1016/j.ajhg.2010.04.005 20471002 PMC3032073

[B5] Almasy LA Dyer TD Peralta JM Kent JW Jr Charlesworth JC Curran JE Blangero J Genetic Analysis Workshop 17 mini-exome simulation BMC Proc 2011 5 suppl 9 S2 22373155 10.1186/1753-6561-5-S9-S2 PMC3287854

[B6] Chen GK Thomas DC Using biological knowledge to discover higher order interactions in genetic association studies Genet Epidemiol 2010 34 863 878 10.1002/gepi.20542 21104889

[B7] Besag J York J Mollie A Bayesian image restoration, with two applications in spatial statistics Ann Inst Stat Math 1991 43 1 20 10.1007/BF00116466

[B8] Kass RE Raftery AE Bayes factors J Am Stat Assoc 1995 90 773 795 10.2307/2291091

[B9] Dering C Pugh E Ziegler A Statistical analysis of rare sequence variants: an overview of collapsing methods Genet Epidemiol 2011 X suppl X X X 10.1002/gepi.20643 PMC3277891 22128052

[B10] Tibshirani R Regression shrinkage and selection via the Lasso J R Stat Soc Ser B Stat Methodol 1996 58 267 288

[B11] Zhou H Sehl ME Sinsheimer JS Sobel EM Lange K Association screening of common and rare genetic variants by penalized regression Bioinformatics 2010 26 19 2375 82 10.1093/bioinformatics/btq448 20693321 PMC3025646

